# A190 EPIDEMIOLOGY OF MUSCULOSKELETAL MANIFESTATIONS IN PAEDIATRIC INFLAMMATORY BOWEL DISEASE: A SYSTEMATIC REVIEW

**DOI:** 10.1093/jcag/gwab049.189

**Published:** 2022-02-21

**Authors:** A Ali, M Schmidt, D Piskin, E Crowley, R Berard

**Affiliations:** 1 Paediatrics, Western University Schulich School of Medicine & Dentistry, London, ON, Canada; 2 Lawson Health Research Institute, London, ON, Canada; 3 Pediatrics, Victoria Hospital & Children’s Hospital, London, ON, Canada

## Abstract

**Background:**

Paediatric inflammatory bowel disease (p-IBD) is a chronic and relapsing gastrointestinal disorder of childhood with associated long-term morbidity. Several extraintestinal manifestations (EIMs) are described, the most common being joint pain and/or inflammation. In 1986, Passo et al. were the first to describe the association of arthritis in p-IBD patients. However, since then, data on the epidemiology, patient and disease factors associated with, treatments for, and outcomes of p-IBD associated musculoskeletal (MSK) disease are not well-established.

**Aims:**

Our study aims to summarize the literature on the epidemiology of MSK EIMs in p-IBD in the era of biologics.

**Methods:**

A systematic review of the literature was performed. PubMed, Embase, Cochrane Library, Web of Science Core Collection, and CINAHL databases were searched with relevant keywords. Studies in English published from January 1, 2000 to December 21, 2020 were included. In total, 3893 papers were identified and screening was performed by two independent reviewers (AA, MS). Conflicts were resolved by a third reviewer (EC, RB). Study and population characteristics were recorded. The primary outcomes of interest were MSK symptoms at presentation and their course, method of diagnosis and definitions used for MSK EIMs. Risk of bias assessment was performed using the JBI Critical Appraisal Tools.

**Results:**

Thirteen studies were included for full review, which were primarily single-centre observational studies with retrospective or cross-sectional design. The method of diagnosis for MSK EIMs varied across the studies, with only 4 studies stating the involvement of a rheumatologist in diagnosis. The definitions also varied, with MSK EIMs such as peripheral arthritis, axial arthritis, enthesopathy, and arthralgia included. Only 7 studies focused on MSK EIMs as their primary outcome, while the remainder reported on all p-IBD associated EIMs. There was a wide range in the prevalence of MSK EIMs from 2–35% (Figure 1). Four studies reported on the therapeutic response of MSK EIMs, and only 3 of those reported on biologic use. Risk of bias demonstrated heterogeneity in the quality of included studies.

**Conclusions:**

This is the first systematic review of the literature for MSK EIMs in p-IBD. Analysis was limited due to variability in study design and data-reporting methods. Included studies reported prevalence of MSK EIMs, but the ascertainment of MSK EIMs, both method and definition varied with a clear lack in standardization. Our study demonstrates the need for further research of the MSK associations of p-IBD and to explore optimal management to advance care for this group of children.

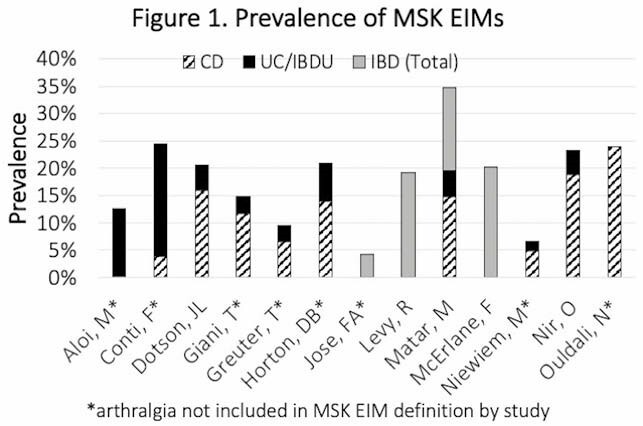

**Funding Agencies:**

None

